# *Plesiomonas*: A Review on Food Safety, Fish-Borne Diseases, and Tilapia

**DOI:** 10.1155/2021/3119958

**Published:** 2021-09-21

**Authors:** Alejandro De Jesús Cortés-Sánchez, Luis Daniel Espinosa-Chaurand, Mayra Díaz-Ramirez, Erika Torres-Ochoa

**Affiliations:** ^1^Consejo Nacional de Ciencia y Tecnología (CONACYT), Unidad Nayarit del Centro de Investigaciones Biológicas del Noroeste (UNCIBNOR+), Calle Dos, No. 23, Cd. del Conocimiento, Av. Emilio M. González C.P., Tepic 63173, Nayarit, Mexico; ^2^Universidad Autónoma Metropolitana, Unidad Lerma, Departamento de Ciencias de la Alimentación, División de Ciencias Biológicas y de la Salud, Av. de las Garzas 10, Col. El panteón, C.P. 52005, Lerma de Villada, Estado de México, Mexico; ^3^Universidad Autónoma de Baja California Sur, Departamento Académico de Ingeniería en Pesquerías, Carretera al sur Km 5.5. Colonia el Mezquitito, C.P. 23080, La Paz, Baja California Sur, Mexico

## Abstract

Fish and fish products are considered a fundamental part of the human diet due to their high nutritional value. Food-borne diseases are considered a major public health challenge worldwide due to their incidence, associated mortality, and negative economic repercussions. Food safety is the guarantee that foods will not cause harm to the health of those who consume them, and it is a fundamental property of food quality. Food safety can be at risk of being lost at any stage of the food chain if the food is contaminated by pathogenic microorganisms. Many diverse bacteria are present in the environment and as part of the microbiota of food that can be transmitted to humans during the handling and consumption of food. *Plesiomonas shigelloides* has been mainly associated with outbreaks of gastrointestinal diseases due to the consumption of fish. This bacterium inhabits the environment and aquatic animals and is associated with the microbiota of fish such as tilapia, a fish of importance in fishing, aquaculture, commercialization, and consumption worldwide. The purpose of this document is to provide, through a bibliographic review of databases (Scopus, Web of Science, and Google Scholar, among others), a general informative perspective on food-borne diseases and, in particular, the consumption of fish and tilapia. Diseases derived from contamination by *Plesiomonas shigelloides* are included, and control and prevention actions and sanitary regulations for fishery products established in several countries around the world are discussed to promote the safety of foods of aquatic origin intended for human consumption and to protect public health.

## 1. Introduction

Feeding is an essential act in the life of humans. However, it is essential that the food to be consumed is safe and provides the necessary nutrients to perform basic functions (energetic, structural, functional, and regulatory) and maintain health [1, 2].

Foods can be disease transmitters, as foods can be contaminated by different microorganisms and/or toxins that, when ingested, become potentially dangerous. Thus, food safety has become an elementary property that involves public health, the well-being of the population, and the world economy [1].

In recent years, fish meat has been considered an alternative to the consumption of beef, pork, and poultry due to the demand for a healthier lifestyle [1]. In the search to satisfy the growing demand for fish, food safety is an essential element to consider since these animals can be vehicles for the transmission of various pathogens, such as *Salmonella* spp.*, Vibrio* spp*., Aeromonas* spp.*, Campylobacter* spp., *Shigella* spp*., Yersinia* spp.*, Clostridium* spp.*, Bacillus cereus, Escherichia coli, Listeria monocytogenes, Staphylococcus aureus,* and *Plesiomonas shigelloides*, among others, which have been responsible for numerous cases and outbreaks of food-borne diseases in humans worldwide [3–6].

In foods such as fish, the presence of a high content of water and nutrients (proteins, lipids, vitamins, and minerals), along with a pH close to neutrality and warm temperatures, provides a favorable environment for rapid microbial growth [5, 7]. Microorganisms can inhabit and survive in many natural environments and can be transferred to food, contaminating them at different stages of the food chain through soil, air, water, insects, animals, and humans [3, 5, 8]. The absence of or inadequate hygienic conditions and practices in production, processing, and conservation favor the transfer of pathogenic microorganisms to food, giving rise to important health risks to the consumer [5, 7–9].

In this context, the purpose of this document is to provide, through a bibliographic review of different databases (Scopus, Web of Science, Redalyc, SciELO, and Google Scholar, among others), a general informative perspective related to members of the food chain (producers, processors, handlers, academia, and the general public) on diseases transmitted by food and, in particular, fish and tilapia, where tilapia is considered an important product in capture fisheries and aquaculture activities with worldwide commercialization and consumption. Food-borne diseases resulting from contamination by *Plesiomonas shigelloides*, control and prevention actions such as the implementation of the Hazard Analysis and Critical Control Points (HACCP) system, microbiological analysis, and the sanitary regulations established in several countries around the world for fish and fish products are specifically considered to promote the safety of foods of aquatic origin intended for human consumption and to protect public health.

## 2. Food-Borne Diseases

Food safety guarantees that a food will not cause harm or disease to the person who consumes it, and it is considered, along with the nutritional, sensory, and commercial characteristics, a component of the total quality of a food [10].

A food-borne disease (FD) is defined as a syndrome caused by the ingestion of food and/or water with etiological agents that affect the health of the consumer [11]. Food-borne diseases present a variety of gastrointestinal symptoms, such as nausea, vomiting, diarrhea, abdominal pain, and fever, and in some cases, they may have complications such as sepsis, meningitis, abortions, Reiter's syndrome, Guillain–Barré syndrome, cancer, or death [8, 11]. These diseases affect mostly children, pregnant women, people with compromised immune systems, and the elderly [11]. Estimations from the World Health Organization (WHO) indicate that 1 in 10 people are affected annually by these diseases through food worldwide, with 420,000 deaths, especially in children [12]. Thus, FDs are considered a public health challenge worldwide due to their morbidity and mortality, as well as their negative effects on productivity, trade, costs in health services, implementation, and monitoring of food safety [7, 11, 13, 14].

The factors that have been related to an increase in the incidence of these diseases are changes in lifestyle, changes in eating habits, socioeconomic level, and aging of the population, among others [14, 15]. Approximately 250 causative agents of FD have been described, including metals, toxins, bacteria, viruses, fungi, parasites, and prions [14]. Among these, bacteria are mostly related to cases and outbreaks [9, 11].

## 3. Fish and Tilapia

In a generic way, “fish” (fish, crustaceans, mollusks, and algae, among others) refers to all food extracted from oceanic or continental waters that can be used for human or animal nutrition [16].

Fish is considered a food of high nutritional value and an essential part of the human diet, as it is a source of proteins of biological value, unsaturated lipids, vitamins, and minerals [16, 17]. The origins of the fish destined for human consumption are capture fishing and aquaculture. These activities reported a worldwide production of 178.5 million tons in 2018, with a per capita consumption of 20.5 kg [18]. Currently, fish is marketed for consumption in different presentations, be it whole, gutted and/or fresh fillet, refrigerated, frozen, modified atmosphere packaging, canned, smoked, salted, dehydrated, and derived products (surimi). [19–21].

However, fish are highly susceptible to deterioration and contamination, and processes of autolysis, oxidation, and microbiological activity intervene in deterioration and affect quality [16, 17]. The quality, nutritional value, and safety of the fish are related to the species, age, habitat, type of feeding, capture conditions, storage conditions, handling, transport conditions, and distribution (Table 1) [17].

*Tilapia*, one of the finned fish with the highest global production, mainly through aquaculture, comprises 10.3% of the total production, with 5,555.4 thousand tons [18]. Through capture fisheries in continental waters, within the 4 groups of species that represent 85% of the total fishing worldwide, tilapia and other cichlids represent the second largest production group, behind carp, barbel, and other cyprinids, with stable catches between 700,000 and 850,000 tons per year, the main producers being various countries in Asia (China, India, and Bangladesh, among others) and Africa (Nigeria, Egypt, Chad, Kenya) [18]. *Tilapia* is a teleost fish of the *Perciforme* order of the *Cichlidae* family, native to Africa, that inhabits most of the tropical regions of the world and that, according to parental care patterns, is classified into three genera: *Tilapia, Oreochromis*, and *Sarotherodon* [26, 27]. Species of the genus *Oreochromis* (*O. niloticus, O. aureus, O. mossambicus*) and their interspecific hybrids (red tilapia) are generally cultivated since these fish can tolerate high densities, their growth is fast, they are resistant to diseases and adaptable to captivity, and they accept balanced feeding diets, in addition to the fact that meat is of high quality and can be sold at an affordable price, making it one of the aquaculture products with the greatest international commercialization, either as a complete product or fillet [26].

### 3.1. Fish Microbiology

The microorganisms in fish, both in free-living and captured specimens, are normally present in the skin (10^2^–10^7^ CFU/cm^2^) and in gills and intestines (10^3^ and 10^9^ CFU/g) and have preponderant effects on quality, deterioration, and safety [28]. The pathogenic bacteria present in fish that are important in the deterioration and safety of fish can be classified into two groups. Group 1: native bacteria, which are widely distributed in aquatic environments around the world, where water temperature has a selective effect. Among these are *Clostridium botulinum, Listeria monocytogenes, Vibrio* sp.*, Aeromonas hydrophila*, and *Plesiomonas shigelloides*. Group 2: nonautochthonous bacteria that are found in fish that are derived from fecal contamination of natural waters or aquatic environments or of the products during their elaboration. Some examples of these microorganisms are *Staphylococcus aureus, E. coli, Salmonella* spp., and *Shigella* spp. and other enterobacteria [4]. In the case of tilapia microbiota, various studies around the world have reported the presence of mainly Gram-negative microorganisms, many of which are pathogens for humans, such as *Aeromonas* spp., *Plesiomonas shigelloides, Shewanella putrefaciens, Pseudomonas* spp*., Vibrio* spp.*, Citrobacter freundii,* and *Escherichia coli*, as well as different Gram-positive microorganisms, such as *Bacillus* spp., *Streptococcus* sp., and *Staphylococcus* spp. [29–32].

## 4. *Plesiomonas*

The genus *Plesiomonas* is made up of a single species, *Plesiomonas shigelloides*, a Gram-negative bacillus belonging to the *Enterobacteriaceae* family, which has the following characteristics and functions: it is 0.3 to 1.0 *μ*m in diameter by 2 to 3 *μ*m in length; it has a genome size of 3.4 Mbp; it has a G + C content is 51% (mol%); it is chemoorganotrophic with a respiratory and fermentative metabolism; it is facultative anaerobic; it is nonsporulated; it is mobile with a polar flagella; it presents somatic antigens “O” and flagellar “H” and recognizes 102 O and 51 flagellar H antigens; it produces the enzymes oxidase, catalase, and tryptophanase; it does not hydrolyze starch; it ferments inositol, glucose, and some other carbohydrates without producing gas; it decarboxylates lysine and ornithine; it mediates arginine dihydrolysis; and it is sensitive to vibriostatic O/129 [27, 32–39].

The bacterium has a growth temperature between 8°C and 45°C, with an optimum temperature between 25°C and 38°C and an optimum growth pH in the range of 4 to 9, and can grow in salt concentrations ranging from 0 to 4% [34–39].

*P. shigelloides* has been isolated from the intestinal contents of humans and animals. Likewise, it is a common inhabitant of aquatic environments, especially in tropical and subtropical regions, including fresh and salt water. Therefore, it can be incorporated in various food products, being found in fresh and salty fish and shellfish; water and food are considered transmission vehicles [29–31, 33–40]. Thus, the consumption of raw or undercooked fish and shellfish is considered one of the main risk factors for infection [27, 36, 40]. Likewise, *P. shigelloides* can cause infections in humans by being present in and transported by untreated water used for drinking, water in recreational facilities, or water used to wash food that is consumed without cooking or heating [34].

Virulence factors that promote disease by this microorganism in humans and animals include the presence of exopolysaccharides for biofilm formation, hydrophobic characteristics of the bacterial surface, adhesion to the host cell surface through fimbriae, mobility through flagella, glycocalyx, DNase, gelatinase, and elastases that allow bacteria to break down proteins of the cell matrix, leading to tissue invasion and spread of infection, enterotoxins, cholera-like toxins, endotoxins (lipopolysaccharide structure, highly immunogenic), cytotoxins, cardiotoxins, *β*-hemolysins, hemagglutinins, iron acquisition systems, and the presence of a plasmid >120 mDa that assists in cell invasion [31–36, 41–43].

The disease caused by *P. shigelloides* is gastroenteritis, and the infectious dose is estimated to be greater than 10^6^ CFU. Ingestion does not always cause this disease, which is generally self-limited and accompanied by fever, chills, abdominal pain, nausea, diarrhea, and/or vomiting; in severe cases, the diarrheal stools can be yellowish green, foamy, and bloody. *P. shigelloides* is also capable of causing septicemia with a high mortality rate and other extraintestinal infections, such as cellulitis, urinary tract infections, peritonitis, meningitis, conjunctivitis, endocarditis, pneumonia, arthritis, endophthalmitis, and cholecystitis [27, 34–36, 44, 45].

The prevalence of *P. shigelloides* gastroenteritis is variable between geographic regions, with lower rates in North America and Europe and higher estimates in Southeast Asia and Africa. In addition, there is a general underestimation of *Plesiomonas* infections commonly because it shares clinical manifestations with other pathogens. Additionally, *P. shigelloides* is not considered for routine analysis in the clinical setting; thus, pathogenic knowledge is limited [46].

In various countries worldwide, such as Japan, Mexico, the USA, Cameroon, Ecuador, Thailand, Nigeria, Cuba, and China, *P. shigelloides* is a clinically relevant pathogen, with numerous cases and outbreaks of diarrhea and gastroenteritis reported that cover all ages ranging from <5 to > 60 years. Infections present a seasonal aspect, where the most reported cases are in the warm months of the year when freshwater temperatures increase, allowing for greater microbial growth and contamination of the waters. In addition, inadequate hygiene practices in food preparation and consumption of untreated water or raw or undercooked fish and shellfish prior to illness showed causal relationships [1, 36, 46–49].

The incidence of health-related *Plesiomonas* infections in immunosuppressed people has been reported to be increasing, especially considering current lifestyles. It is estimated that climate change and global warming promote an increase in the incidence of infectious diseases (including *Plesiomonas*) transmitted mainly by water [46].

Treatment for infection by *Plesiomonas shigelloides* generally consists of oral rehydration, while in severe cases, the use of antimicrobials such as norfloxacin, trimethoprim, azithromycin, ceftriaxone, or ciprofloxacin is recommended [36, 50]. In recent years, the isolation of *Plesiomonas* from the aquatic environment showing resistance to several antimicrobials, many of which are used in the treatment of infections, has been reported; it has been pointed out that antimicrobial resistance is the product of contamination by human activities, which has become a serious threat to public health and to these reservoir environments [51–53].

### 4.1. *Plesiomonas* and Tilapia

*Plesiomonas* is not considered part of the normal microbiota of humans, although the opposite is true for freshwater fish from aquaculture and the environment, such as tilapia, which are indicated as a primary reservoir, as well as the water and sediment of their habitat. *Plesiomonas* acts as a facultative or opportunistic pathogen in fish, causing epizootic outbreaks associated with stress-promoting factors such as handling, poor hygiene conditions, a high amount of organic matter in ponds, and an increase in water temperature, ultimately triggering high mortality rates and loss in aquaculture productivity [27, 29, 36, 40, 54, 55]. Considering the increases in the fishing and aquaculture production and commercialization of these fish around the world, the focus has been on the safety and control of these products and their prevention since they are a potential source of diseases to humans [40, 54, 56].

## 5. Control and Prevention of Diseases in Humans due to *Plesiomonas shigelloides*

Contamination of food by *P. shigelloides* and subsequent human diseases are generally related to exposure to contaminated water sources along the food chain, as well as to the consumption of raw or undercooked fish and shellfish [35].

For the development and implementation of control and prevention actions, an interdisciplinary orientation is required that involves actors in the food production and supply chain, such as health regulatory authorities, producers, industrialists, and merchants. These actions involve the surveillance and sanitary regulation of products, implementation of good practices in primary production and manufacturing, and the Hazard Analysis and Critical Control Points (HACCP) system, with the latter being one of the main preventive control measures. Likewise, education and awareness actions on the hygiene and microbiological safety of food are necessary, focused on handlers and final consumers [7, 35, 57].

At a global level, it is important to establish and harmonize the quality and safety of food to contribute to the control and prevention of food-borne diseases due to contamination by different causal agents of biological origin, such as various enterobacteria, including *Plesiomonas*. The Food and Agriculture Organization of the United Nations (FAO) and the World Health Organization (WHO) created the Codex Alimentarius, which is a set of codes of practice, guidelines, and standards on issues related to hygiene, contaminants, labeling, presentation, and additives that are focused on raw, semiprocessed, or processed foods for distribution to the consumer or as raw material. Codex Alimentarius standards are internationally accepted to guarantee food safety and facilitate global trade [58]. In the case of fish and fish product safety, some components of the Codex Alimentarius normative framework include the general principles of food hygiene (CXC 1-1969) [59], the code of practice for fish and fishery products (CXC 52-2003) [60], and standards for quick-frozen finned fish, both uneviscerated and eviscerated (CODEX STAN 36-1981) [61], among others.

Different countries around the world, such as the European community, have established food safety measures for human consumption through different regulations (EC), such as N^o^. 178, 852, 853, 854, and 2073 [62–66], the latter based on microbiological criteria to be met in the production and commercialization of fish and fish products, but these measures lack a section referring to *Plesiomonas shigelloides,* unlike other enterobacteria, such as *Salmonella* spp., and *Vibrio* spp., making it necessary to analyze the incorporation of *P. shigelloides* in the regulatory framework for fish products. Meanwhile, in Latin America, countries such as Mexico have sanitary regulations for fish and fish products through NOM-242-SSA1-2009 [67], a standard in which, unlike *Enterobacteriaceae*, such as *Salmonella* spp., and *Vibrio* spp., which must be absent from the product, *E. coli* should be at a maximum of 400 MPN (most probable number)/g. However, this standard does not present microbiological criteria applicable to *Plesiomonas shigelloides*. The NOM-251-SSA1-2009 standard [68] establishes hygiene practices for the processing of food, beverages, or food supplements and their raw materials to avoid their contamination throughout their processing and involves the implementation of HACCP systems, and the NOM-128-SSA1-1994 standard [69] specifically establishes the application of a system of risk analysis and control of critical points in the industrial plant that processes fish products. For the particular case of fishery products such as refrigerated fresh tilapia and frozen fresh tilapia, the standards NMX-F-578-SCFI-2001 [70] and NMX-F-579-SCFI-2001 [71], respectively, establish product quality specifications for human consumption, including microbiological analyses for evaluation. However, none of the above standards considers the analysis of *Plesiomonas*.

### 5.1. Food Analysis in the Laboratory

The microbiological analysis of food is carried out with main three objectives: quality control and evaluation of shelf life, review of hygiene procedures in handling and production processes, and control and prevention of diseases through food [72]. *P. shigelloides* can be isolated from environmental, clinical, or food samples using various differential and selective culture media such as Pseudomonas-Aeromonas selective (GSP) agar, thiosulfate citrate bile sucrose (TCBS) agar, and inositol-brilliant-green-bile salt (IBB) agar, as well as some culture media generally used for *Enterobacteriaceae* such as Rimler-Shotts agar, MacConkey (MAC), xylose lysine decarboxylase (XLD), or Hektoen (HE) agar; in the latter, the appearance of typical colonies is variable due to its slow fermentation of lactose [34–36, 73, 74].

**Figure 1:**
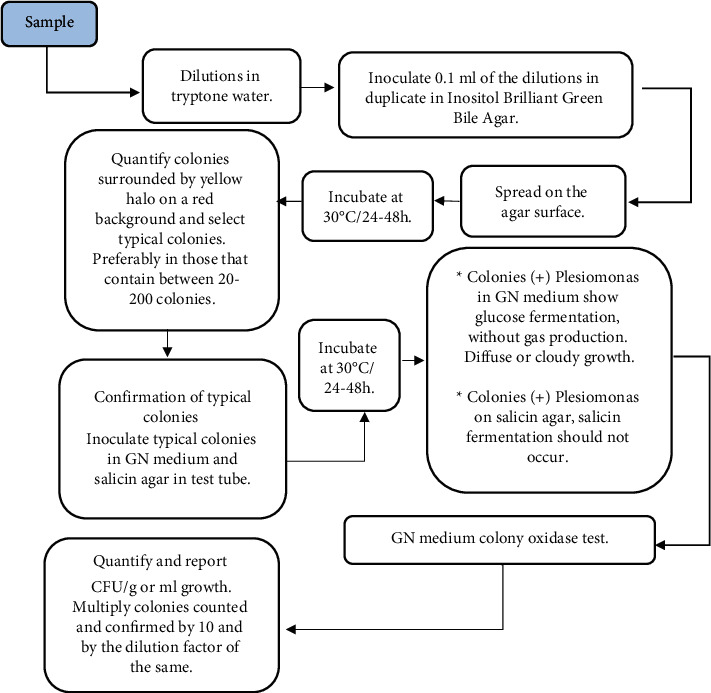
Direct plaque quantification of *Plesiomonas* in food samples [75]. ^∗^GN medium consists of 3 layers of approximately 2 cm each, placed in a sterile culture tube. The bottom layer is bile and red violet glucose agar. The middle layer is agar-water, and the top layer is sulfide indole motility (SIM) agar. ^∗^Glucose fermentation in positive GN medium (turning of the bile and red violet glucose agar medium in the lower layer of the tube). ^∗^*P. shigelloides* is SH_2_- and indole-positive, but these characteristics are not shown in GN medium. ^∗^In GN medium, the mobile species develop, forming diffuse growth along the inoculation streaking line or more intense turbidity.

**Figure 2:**
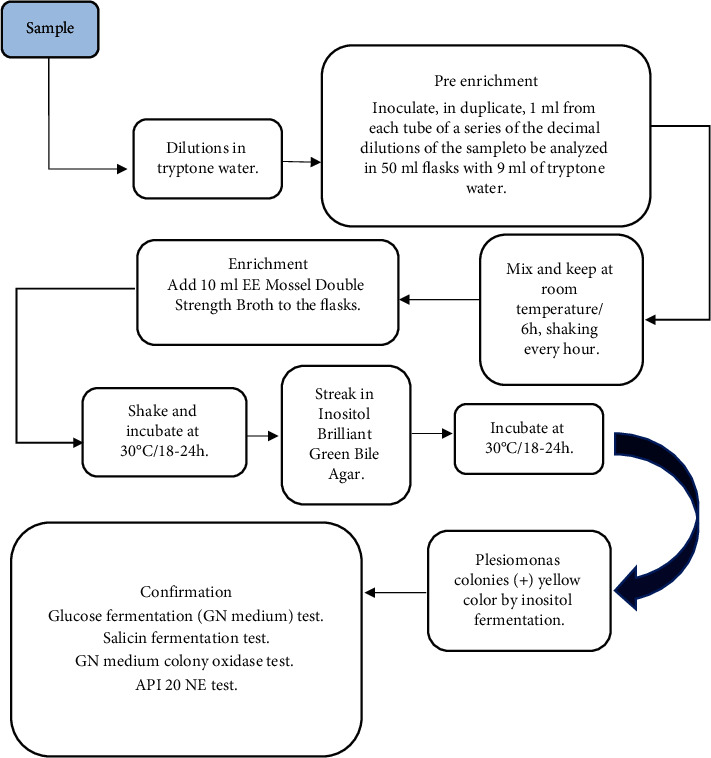
Presence-absence test with enrichment in selective medium [75].

The isolation and detection techniques of *P. shigelloides* in food are based on direct sowing and counting in plates with selective culture medium, generally using a previous enrichment phase with alkaline peptone water pH 8.6, with or without the addition of 1–3% NaCl, bile peptone broth, tetrathionate broth, Mossel EE broth, or others due to the stress to which the microorganism is subjected in the different phases through which the food passes from its production to consumption [34, 36, 73–75]. The culture media generally used for selective isolation are inositol-bright green-bile salt (IBB) agar and cefsulodin-irgasan-novobiocin (CIN) agar [35, 36, 75]. Anderson et al. [75] reported two detection methods for *P. shigelloides*. A direct plate quantification method using sample dilutions and inoculation on the surface of IBB selective agar was performed, and positive inositol fermentation colonies were subsequently selected for confirmation using GN medium (Gram-negative bacteria) and salicin agar as well as different biochemical tests, such as oxidase (Figure 1). Another method reported by researchers to determine the presence or absence of *Plesiomonas* in food samples involves using pre-enrichment media (tryptone water), selective enrichment (EE Mossel broth), and differentials such as IBB agar, as well as biochemical confirmation tests (GN medium, salicin agar, and oxidase). The enrichment phases were preferably incorporated for the detection of the pathogen due to a possible reduced amount present, to the stress to which it was subjected in the food or to inhibit the accompanying microbiota in the sample (Figure 2). Growth inhibition in 6% NaCl and biochemical tests for arginine dihydrolase and l-histidine decarboxylase can be used to differentiate the growth of *P. shigelloides* from *Vibrio* spp., while the tests for lysine decarboxylase and ornithine decarboxylase differentiate *P. shigelloides* from *Aeromonas* spp., and the cytochrome oxidase test differentiates *P. shigelloides* from other *Enterobacteriaceae* (Table 2) [35, 56, 76]. Other biochemical identification tests used include indole, inositol, and glucose fermentation, production of *β*-hemolysis, sensitivity to vibriostatic O/129 or a variety of commercial kits, such as API 20E, the Vitek 2 system, or the BD Phoenix [27, 34, 36].

Within the analysis for the detection of *P. shigelloides* as an alternative to the traditional microbiological analysis, molecular techniques have been developed based on the polymerase chain reaction (PCR) with its different variants, which show greater specificity, sensitivity, reliability, and speed in the detection of the pathogen in fish and tilapia samples, and that use as targets regions of the *23S rRNA gene, 16S rRNA, gyrB*, *fur* (ferric uptake regulator), and *hugA* gene, the latter encoding an outer membrane receptor required for the use of 435-bp heme iron [36, 40, 41, 55, 79]. Likewise, among the molecular tools focused on the genotyping of isolates for phylogenetic and epidemiological studies are multilocus sequence typing (MLST), random amplified polymorphic DNA (RAPD)-PCR, enterobacterial repetitive intergenic consensus (Eric)-PCR, repetitive extragenic palindromic (REP)-PCR, and pulsed field gel electrophoresis (PFGE) [80, 81]. Meanwhile, there are techniques for the identification and phenotypic characterization of bacterial isolates based on the analysis of proteins (mainly ribosomal) through the creation of a specific mass spectrum of genus and species (fingerprint) based on the sensitivity, accuracy, speed, and reproducibility of matrix-assisted laser desorption ionization-time of flight mass spectrometry (MALDI-TOF MS) [36, 82, 83].

## 6. Conclusions

Fish are considered a nutritious food that is widely produced, marketed, and consumed around the world. *Tilapia* is among the fish with the highest production, mainly in aquaculture destined for human consumption. Fish are highly susceptible to contamination and deterioration along the food chain by different microorganisms, such as *Plesiomonas shigelloides*, which is a causal agent of disease in fish, such as tilapia and humans, negatively affecting primary production and food safety. For *P. shigelloides*, which is common in natural and aquacultural water systems and is considered part of the microbiota of freshwater fish such as tilapia, the implementation of good hygiene practices in production, processing and preservation, and sanitary surveillance of regulatory authorities and the food industry, as well as the promotion of hygiene education in the handling and final preparation of these foods for consumption, can reduce the risk of contamination and outbreaks of food-borne diseases.

## Figures and Tables

**Table 1:** Different hazards to the safety of fish and fish products intended for human consumption [22–25].

	Microorganism	Causal agents of food-borne diseases
Hazards	Bacteria	*Vibrio* sp., *Salmonella* spp., *Shigella* sp., *Plesiomonas shigelloides, Edwardsiella tarda, Listeria monocytogenes, Staphylococcus aureus, Escherichia coli, Clostridium botulinum, Clostridium perfringens, Bacillus cereus, Campylobacter jejuni, Aeromonas hydrophila.*
	Fungi	*Fusarium* spp., *Aspergillus* spp.*, Penicillium* spp.
	Viruses	Hepatitis A, hepatitis E, adenovirus, norovirus, astrovirus, rotavirus, enterovirus.
	Parasites	*Gnathostoma* sp.*, Pseudoterranova* sp.*, Anisakis* sp.*, Phocanema* spp*., Angiostrongylus* sp.*, Contracaecum* sp., *Diphyllobothrium* sp., *Phagicola* sp., *Clonorchis* sp., *Paragonimus* sp., *Heterophyes* sp., *Cryptosporidium* sp.
	Chemical compounds	
	Biotoxins	Tetrodotoxin, ciguatera (ciguatoxin, scaritoxin, maitotoxin, palytoxin, and okadaic acid), gempilotoxin, and mycotoxins
	Heavy metals	Lead, cadmium, copper, mercury
	Organic compounds	Polycyclic aromatic hydrocarbons, polychlorinated biphenyls, polybrominated diphenyl ethers, dioxins, pesticides, microplastics, antibiotics, and hormones
	Nitrogen compounds biogenic amines	Histamine, putrescine, and cadaverine by the decarboxylation of histidine, ornithine, and lysine, respectively, in activities mediated by bacterial metabolism

**Table 2:** Biochemical identification among *Plesiomonas, Vibrio*, and *Aeromonas* bacteria present in fish and fish products [29–32, 75–78].

Test	Microorganism		
	*Vibrio*	*Aeromonas*	*Plesiomonas*
Oxidase	+	+	+
Mobility	+	+	+
Indole	+	+	+
Mannitol	+	+	−
O129	S	R	R
LDCA	+	−	+
ODCA	(+)	−	+
ADHA	−	(+)	+
Inositol	−	−	(+)

+: positive, −: negative, (+): late positive, S: sensitive, R: resistant. LDCA: lysine decarboxylase, ODCA: ornithine decarboxylase, ADHA: arginine dihydrolase, vibriostatic agent O/129.
